# Supplementary material for: Neural mechanisms of transitive inference and fast knowledge reassembly in learning neural networks
Source: bioRxiv. 2023 Jul 28:2023.07.27.550739. Preprint. [Version 1] doi: 10.1101/2023.07.27.550739 (PMC10402151; doi:10.1101/2023.07.27.550739)
Supplement: Supplementary file 1 [file NIHPP2023.07.27.550739v1-supplement-1.pdf]

bioRxiv preprint doi: https://doi.org/10.1101/2023.07.27.550739; this version posted July 28, 2023. The copyright holder for this preprint (which was not certified by peer review) is the author/funder, who has granted bioRxiv a license to display the preprint in perpetuity. It is made available under aCC-BY 4.0 International license.

A Learning multiple lists

We ensure that the networks can learn multiple orderings rather than just one. This is done with two interventions. First, we only reset plastic weights $\mathbf{P}(t)$ every third episode. Second, the "test" period of each episode (the last 10 trials) include 25% of pairs involving stimuli from the previous ordering since the last $\mathbf{P}(t)$ reset (if any). While not immediately relevant to the present work, this constitutes a simple example of meta-continual learning, where the goal is not just to be able to learn new problems efficiently, but also to keep in memory previously seen problems, at least up to a short horizon. Performance on pairs from previous lists was consistently high (above 70%), confirming that the model is able to learn and store several different lists.

The above procedure was followed for all training. At test time, among the results reported in this paper, the list-linking examples are implemented as three successive episodes of different lengths (plus a single test trial), without initialization of $\mathbf{P}(t)$ between these episodes. All other reported results used a single test episode with $\mathbf{P}(t)$ initialized to 0 at the start of the episode.

B Meta-Reinforcement Learning algorithm: The A2C algorithm

The outer loop of our method ascends the gradient of episodic reward over the network's structural parameters (the various $\mathbf{W}$ matrices and $\mathbf{A}$, plus additional scalar parameters; see below). Gradients are estimated by A2C (Advantage Actor-Critic), a well known reinforcement learning (RL) algorithm that is commonly used in meta-RL experiments [Duan et al., 2016, Wang et al., 2016, Miconi et al., 2018, Wang et al., 2018].

First, in addition to previously discussed outputs, the network also produces a scalar output $\hat{v}$ (the "value-prediction" output), through the weight vector $\mathbf{w}_{\hat{v}}$. This output tries to predict, at each point in time, the current value of $V(t) = \sum_{t'=t}^{T_{max}} \gamma^{t'-t} R(t')$, that is, the sum of future discounted rewards during the episode.

After the episode concludes, we retroactively go back through the history of rewards obtained at each time step, allowing us to compute the real, ground-truth $V(t)$ for each time step. Then the actual objective J to be maximized is computed as follows:

$$J = \sum_{t=0}^{T_{max}} \beta_1(t)\, \pi_t(a_t)\, \mathrm{StopGradient}(V(t) - \hat{v}(t)) + \beta_2 H(\pi_t) - \beta_3 (V(t) - \hat{v}(t))^2 \tag{8}$$

Here, a_t is the action (i.e. the response) given at time t, π_t is the probability distribution over both possible actions/responses (determined at each time step by a SoftMax over both output neurons of the network, see Methods). H denotes a measure of dispersion of a probability distribution, usually implemented as the Shannon entropy; in practice, due to computational limitations, we set the H term as the sum of squares (an alternative measure of dispersion) rather than the actual Shannon entropy. $\beta_1(t)$ is a multiplier set to 1 for the first 20 trials (on adjacent pairs only) and 4 for the last 10 trials (on all pairs; see Methods), and β_2, β_3 are positive constants. Note that the second term encourages the output distribution over possible actions to be as uniform as possible (with the goal of favouring exploration), while the third term optimizes the value predictor head $\hat{v}(t)$.

Intuitively, this algorithm can be thought of as a version of the standard REINFORCE method for estimating the gradient of rewards over weights that control stochastic responses: take the weight modification that would make the response you just gave more likely in the future for the same inputs, and multiply it by the actual rewards obtained. Furthermore, in A2C (as in other actor-critic methods), the actual rewards are centered by subtracting a network-generated estimate of future discounted rewards $\hat{v}(t)$. This centering has

bioRxiv preprint doi: https://doi.org/10.1101/2023.07.27.550739; this version posted July 28, 2023. The copyright holder for this preprint (which was not certified by peer review) is the author/funder, who has granted bioRxiv a license to display the preprint in perpetuity. It is made available under aCC-BY 4.0 International license.

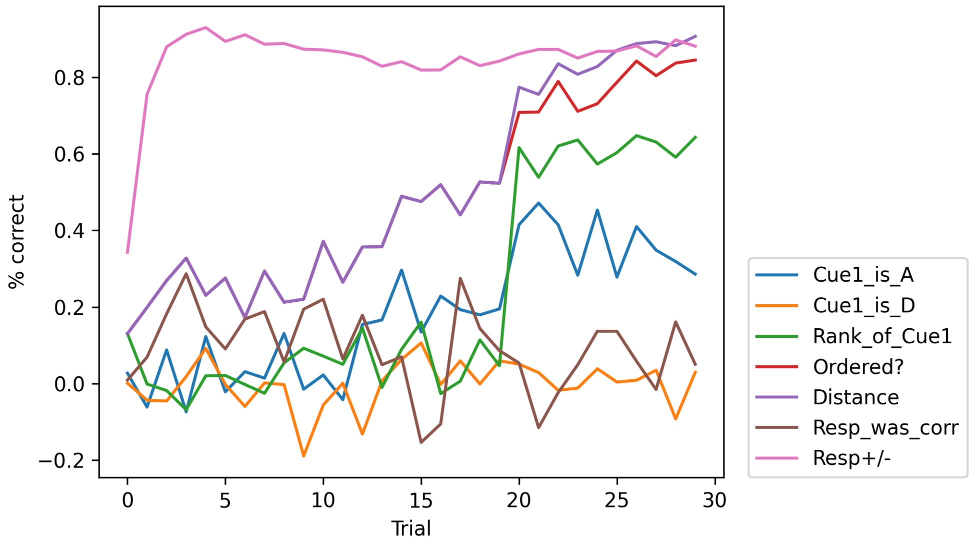

Figure S1: Decoding performance, at each trial in the episode, for various quantities. See text for details.

the effect of reducing the variance in the gradient estimate. In addition to this, training the value predictor also plays the role of an "auxiliary loss": a signal with a hard ground truth that provides information about environment structure. Finally, A2C also adds an $H(t)$ term to encourage maximally uniform distribution over possible outputs, and thus (hopefully) exploration.

The parameters being subject to gradient descent between episodes are $\mathbf{W}$ matrices, $\mathbf{A}, \eta$, and a scalar multiplier on $m(t)$. Gradients of J over these parameters are computed automatically by PyTorch, using the Adam optimizer. Note that gradient computation involves backpropagation of gradients through time, since activities at time t influence activities at future times. One step of gradient descent is applied after each episode. We reiterate that no gradient descent occurs during a given episode.

C Individual item rank is not directly encoded in neural representation of pairs

We run a full episode with a trained network over 2000 runs (each with different randomly generated stimuli), and use a linear decoder to decode various quantities from $\mathbf{r}(t = 2)$ (step 2 neural activations), separately for each trial. We train our decoder on data from the first 1800 runs, and test it on data from the last 200 runs.

Results are shown in Figure S1. The quantities we try to decode are:

- *Cue1_is_A*: whether item 1 in this trial is A.
- *Cue1_is_D*: whether item 1 in this trial is D
- *Rank_of_Cue1*: the actual numerical rank of item 1

bioRxiv preprint doi: https://doi.org/10.1101/2023.07.27.550739; this version posted July 28, 2023. The copyright holder for this preprint (which was not certified by peer review) is the author/funder, who has granted bioRxiv a license to display the preprint in perpetuity. It is made available under aCC-BY 4.0 International license.

- *Ordered?* whether the items were shown in descending order (Stim1 ¿ Stim2) in this trial
- *Distance*: the distance in rank between the items (which is always 1 for the first 20 trials)
- *Resp_was_corr*: whether the network's response was correct for this trial
- *Resp+/-*: the network's response for this trial

Information about the network's response is clearly encoded in $\mathbf{r}(t = 2)$ from early on in the episode (pink curve). Information about the ordering of the two items (i.e. the actual correct response for this trial) and their distance slowly increases over time (purple and red items). Note that before trial 20, all pairs shown are adjacent pairs, and thus item ordering and item distance (always -1 or +1) carry the same information, explaining their identical curves up to this point.

By contrast, information about the identity of each individual item is not encoded at all before trial 20. Even after trial 20, the apparent ability to decode item 1 rank is very similar to that of a classifier only given the correct response for this trial as input; that is, $\mathbf{r}(t)$ does not seem to possess additional information about individual item rank above and beyond that provided by knowledge of the correct response for this trial (data not shown). Thus, the network does not seem to reliably encode individual item identity in its responses.

D Representation changes for item pairs other than DE

In Figure S2, we show step-by-step representation changes at trial 20 when trial 20 involved the pair CD (top) or EF (bottom), rather than DE as in Figure 8. We see that results are very similar: representation changes occur at time step 4, and involve not just the items shown at this trial, but also adjacent items (B and E, or D and G, respectively).

E Transfer of representation changes to unseen neighbouring items requires previous joint presentation

In Figure S3, we plot the same measurements as in Figure 8. However, instead of including all runs in which the model did not observe item pair DE or ED before trial 20, we now only include runs in which the model observed neither DE/ED nor CD/DC (item pair shown at trial 20 is still DE or ED). Figure S3 shows that for those runs, no transfer to item C occurs: at step 4 of trial 20 (when the model first observes pair DE/ED), items D, E and F see some change in their alignment with the decision axis, but item C does not.

Figure S4 shows the same experiment but now the additional withheld pair is EF instead of CD. Now learning does not transfer to item F, as expected. This confirms that transfer between neighbouring items only occurs if they have been shown together before.

F Identifying recoded representations with an optimization procedure

Our analysis indicated that representation learning occurred at time step 4, and involved a shift in the step-2 representation of relevant items along the axis of the output weight vector (with either sign) (Figure

bioRxiv preprint doi: https://doi.org/10.1101/2023.07.27.550739; this version posted July 28, 2023. The copyright holder for this preprint (which was not certified by peer review) is the author/funder, who has granted bioRxiv a license to display the preprint in perpetuity. It is made available under aCC-BY 4.0 International license.

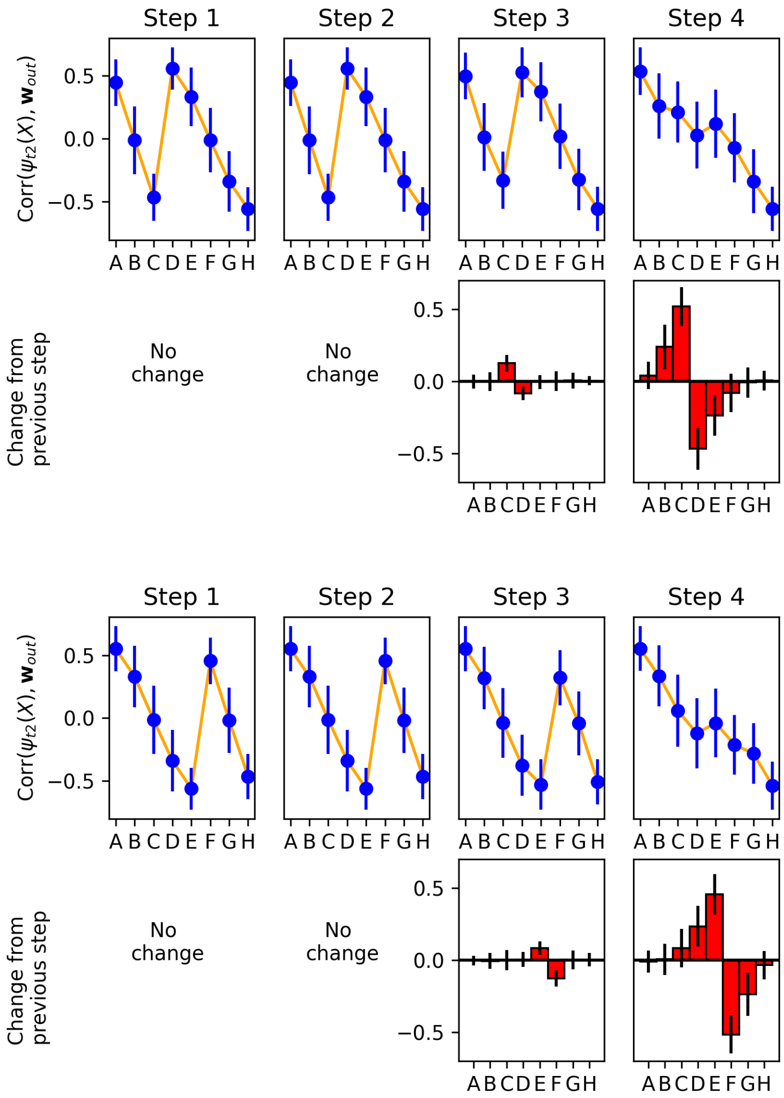

Figure S2: Same as Figure 8, but setting the pair first shown on trial 20 to either CD (top) or EF (bottom) rather than DE. Learning proceeds similarly for these other pairs.

bioRxiv preprint doi: https://doi.org/10.1101/2023.07.27.550739; this version posted July 28, 2023. The copyright holder for this preprint (which was not certified by peer review) is the author/funder, who has granted bioRxiv a license to display the preprint in perpetuity. It is made available under aCC-BY 4.0 International license.

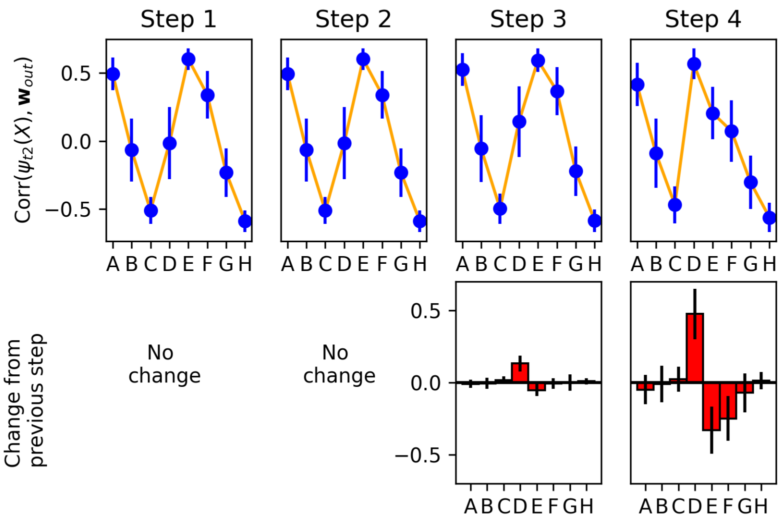

Figure S3: Changes in alignment at each step of trial 20, when the model sees DE (in any order) at trial 20, but saw neither DE nor CD (in any order) before. At time step 4, no transfer of learning to C occurs.

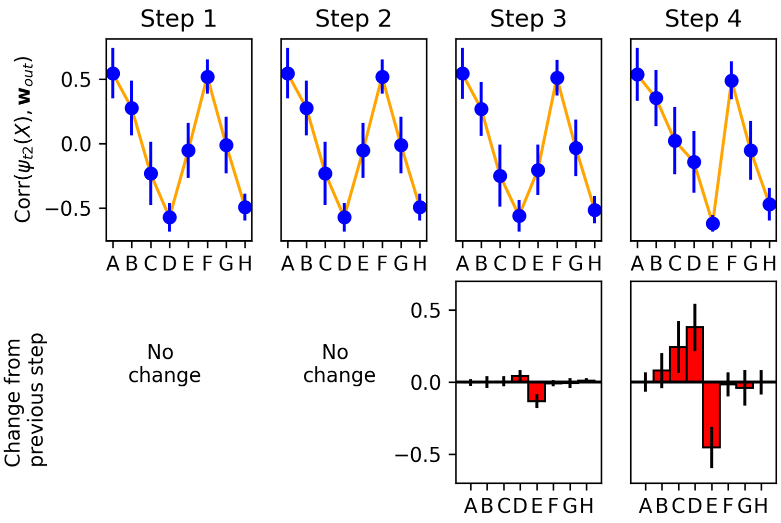

Figure S4: Same as Figure S3, but the additional withheld pair (in addition to DE) is now EF instead of CD. Now, at time step 4, no transfer of learning to F occurs.

bioRxiv preprint doi: https://doi.org/10.1101/2023.07.27.550739; this version posted July 28, 2023. The copyright holder for this preprint (which was not certified by peer review) is the author/funder, who has granted bioRxiv a license to display the preprint in perpetuity. It is made available under aCC-BY 4.0 International license.

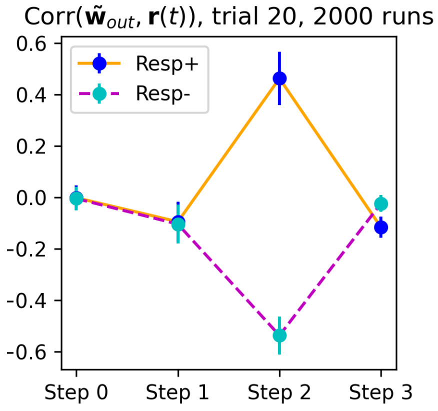

Figure S5: Correlation between $\mathbf{r}(t)$ and $\tilde{\mathbf{w}}_{out}$, at each time step of trial 20 (2000 runs), shown separately for runs in which network response at trial 20 was positive vs. negative. The adapted representation of the output weight vector $\tilde{\mathbf{w}}_{out}$ is strongly represented at step 3, with sign correlated with network response for this trial.

8). These step-2 representations, in turn, are the result of passing step-1 feedforward representation through one step of recurrence with the learned plastic weights $\mathbf{P}(t)$. Thus, if the step-2 representations change, it implies that $\mathbf{P}(t)$ also changed. How did this change happen?

Recall that plastic weights are shaped by Hebbian learning, which essentially amounts to $\boldsymbol{\Delta}\mathbf{P}(t) \approx m(t)\,\mathbf{x}(t-1)\,\mathbf{r}(t-2)^{\mathsf{T}}$ (see Equation 7). For intuitive understanding, let us overlook the nonlinearity and assume a positive $m(t=4)$. If weight changes were purely Hebbian, including the step-1 representations in $\mathbf{r}(t=2)$ and the output weight vector in $\mathbf{r}(t=3)$ would increase plastic weights at time $t=4$ by $\mathbf{w}_{out}\boldsymbol{\psi}_{t1}^{\mathsf{T}}$, which would have exactly the appropriate effect for representation learning: future presentations of $\boldsymbol{\psi}_{t1}^{\mathsf{T}}$ to the recurrent network (at $t=1$)would produce an output ($\mathbf{r}(t=2)$) more similar to $\mathbf{w}_{out}$ (vs. less similar for a negative $m(t)$). This would constitute a straightforward example of Hebbian associative learning.

However, as seen in Figure S12, this does not occur. Neural activities at time steps 2 and 3 do not seem to contain step-1 representations of the items, or of the output weight vector. This leads to the following question: what signal in the neural activities at time step 2 and 3 produces the representation changes seen at step 4 in Figure 8 ?

We hypothesized that the network does reinstate representations of the items and decision axis, but under a *recoded* form, which would facilitate learning under the constraints imposed upon the network. One such constraint (we surmised) is heterogeneous plasticity. Recall that the recurrent weights are not purely Hebbian, but are rather the sum of a fixed (non-plastic) $\mathbf{W}_{rec}$ and a weighted plastic component $\mathbf{A} \odot \mathbf{P}(t)$ (see Equation 1). Thus, simple Hebbian learning between $\boldsymbol{\psi}_{t1}$ at time $t-2$ and $\mathbf{w}_{out}$ at time $t-1$ would indeed shift the $\mathbf{P}(t)$ component in the correct direction (towards increasing alignment between $\boldsymbol{\psi}_{t2}$ and $\mathbf{w}_{out}$), but the net effect on the *total* recurrent weights $\mathbf{W}_{rec} + \mathbf{A} \odot \mathbf{P}(t)$ would be more complex and unpredictable. To take an extreme example, if $\mathbf{A}$ were fully-negative and larger than $\mathbf{W}_{rec}$, the actual effect on total weights would be in the opposite direction.

What would be the "ideal" activation vectors $\mathbf{r}(t-2)$ and $\mathbf{r}(t-1)$ that would produce the desired weight change in *total* recurrent weights at time step t? Since no obvious analytical answer is available, we

bioRxiv preprint doi: https://doi.org/10.1101/2023.07.27.550739; this version posted July 28, 2023. The copyright holder for this preprint (which was not certified by peer review) is the author/funder, who has granted bioRxiv a license to display the preprint in perpetuity. It is made available under aCC-BY 4.0 International license.

address this question by an optimization procedure. Recall that the desired change is to shift future step-2 representations of each item towards greater alignment with the output weight vector. Therefore, we seek to find the recoded representations $\tilde{\boldsymbol{\psi}}_{t1}(X)$ and $\tilde{\boldsymbol{w}}_{out}$ which, when represented in network activity at times $t-2$ and $t-1$ (and thus modifying $\mathbf{P}(t)$ proportionally to their outer product, due to Hebbian learning) would cause a net change in the *total* recurrent weights, such that future presentations of the original $\boldsymbol{\psi}_{t1}(X)$ would produce an output more similar to the original $\boldsymbol{w}_{out}$.

We initialize $\tilde{\boldsymbol{\psi}}_{t1}(X)$ (for all items X) and $\tilde{\boldsymbol{w}}_{out}$ to small random vectors, and repeatedly perform the following procedure:

1. Compute the outer product $\tilde{\boldsymbol{W}}_{prod} = \tilde{\boldsymbol{w}}_{out}\tilde{\boldsymbol{\psi}}_{t1}^{\intercal}$
2. Substituting this outer product for the plastic weights $\mathbf{P}(t)$ in the total recurrent weights, recompute the recurrent operation on the original step-1, feedforward representations: $\tilde{\mathbf{y}} = (\mathbf{W}_{rec} + \mathbf{A} \odot \tilde{\boldsymbol{W}}_{prod})\,\boldsymbol{\psi}_{t1}$ (recall that $\mathbf{W}_{rec}$ and $\mathbf{A}$ are fixed within each episode)
3. Compute the alignment between this output and the decision axis $\mathbf{w}_{out}$: $J = \mathrm{Corr}(\tilde{\mathbf{y}}, \mathbf{w}_{out})$
4. Perform one step of gradient ascent of J over $\tilde{\boldsymbol{w}}_{out}$ and $\tilde{\boldsymbol{\psi}}_{t1}$.

This procedure is performed in parallel over the batch of 2000 individuals, each with randomly generated stimuli. Because each run has its separate stimuli, we maintain a separate set of $\tilde{\boldsymbol{\psi}}_{t1}(X)$ for each of the 2000 runs. By contrast, we consider $\tilde{\boldsymbol{w}}_{out}$ as an intrinsic feature of the network, and thus use only one $\tilde{\boldsymbol{w}}_{out}$ for the whole batch. We use the Adam optimizer with learning rate 1e-3 and weight decay 1e-2 for regularization.

It is important to note that these recoded representations were not found by specifically exploring network activity at time steps 2 and 3. Rather, they constitute hypotheses about what learning-appropriate representations at these times "should" look like, at least in part. Clearly many other factors affect the structure of ideal representations, e.g. not interfering with the main task (recall that time step 2 is response time, while time step 3 is reward signal time; the reinstated representations should minimize interference with these important ongoing task signals). From Figures 9, 10, and S5, we get confirmation that these "guessed" representations are adequate probes to detect the reinstatement of adequate representations by the network at these time steps, whereas the original unadapted $\boldsymbol{\psi}_{t1}(X)$ and $\boldsymbol{w}_{out}$ are not (Figure S12).

G Robustness to massed presentation

Some existing models of transitive inference are disrupted when one single pair is shown a large number of times [Jensen et al., 2019]. Following existing practice, we ran the trained network on an extended episode, in which 20 additional trials (all with the pair DE) were inserted between the 20 adjacent-only trials and the 10 all-pair trials. No other modifications were made. Again, we performed 2000 runs, each with independently generated stimuli. As shown in Figure S6, there was little impact on performance, whether on transitive inference, the symbolic distance effect, or the end-item effect.

H An alternative, suboptimal solution

Here we describe results from the alternative, suboptimal solution sometimes found by the system, as discussed in section 5. As shown in Figure S8, this solution uses reward-selective neuromodulation $m(t)$ at reward time, while $m(t = 4)$ seems unrelated to reward.

bioRxiv preprint doi: https://doi.org/10.1101/2023.07.27.550739; this version posted July 28, 2023. The copyright holder for this preprint (which was not certified by peer review) is the author/funder, who has granted bioRxiv a license to display the preprint in perpetuity. It is made available under aCC-BY 4.0 International license.

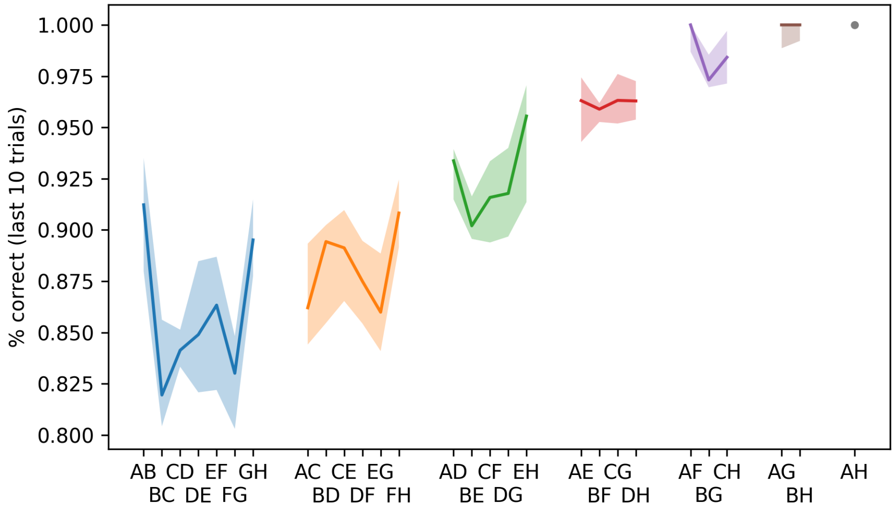

Figure S6: Performance for massed-presentation experiment, grouped by pair. Compare with Figure 3.

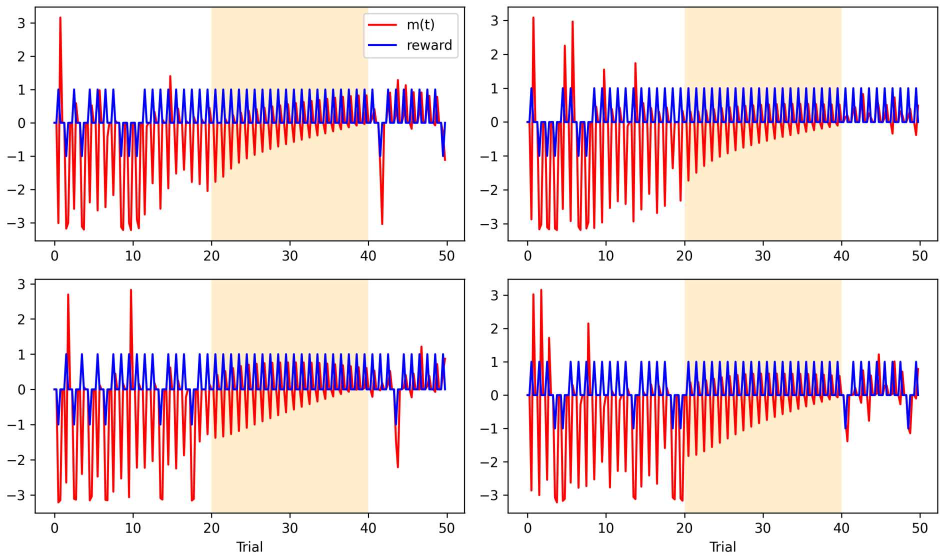

Figure S7: Traces of neuromodulatory output $m(t)$ (red) and reward signal (blue) for 4 episodes with massed presentation of the pair DE between trials 20 and 40.

bioRxiv preprint doi: https://doi.org/10.1101/2023.07.27.550739; this version posted July 28, 2023. The copyright holder for this preprint (which was not certified by peer review) is the author/funder, who has granted bioRxiv a license to display the preprint in perpetuity. It is made available under aCC-BY 4.0 International license.

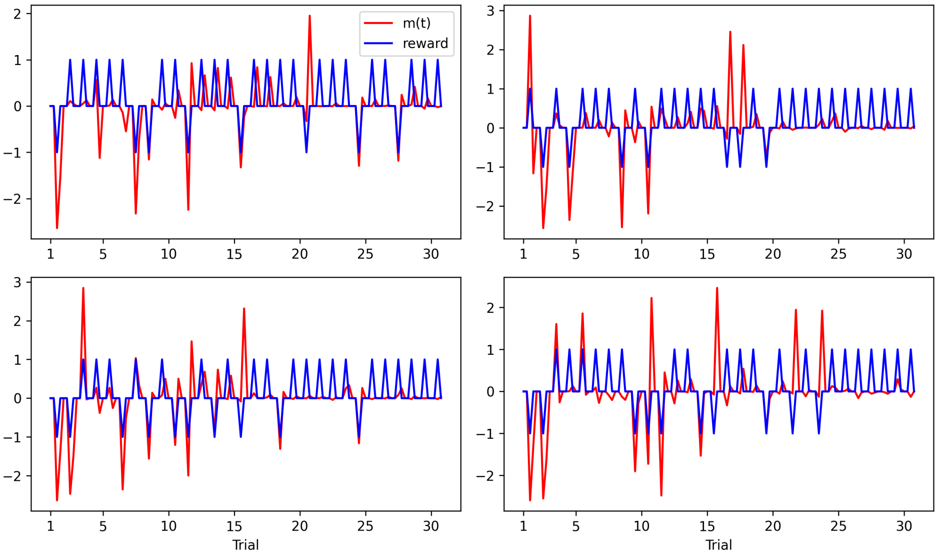

Figure S8: Traces of $m(t)$ (red) and reward signal (blue) over one episode, for four different runs, using a suboptimal network. Conventions are as in Figure 5

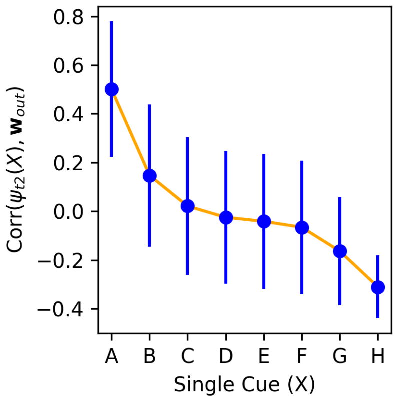

Figure S9: Correlation between the step-2 representation of each item $\boldsymbol{\psi}_{t2}(X)$ and the output weight vector $\mathbf{w}_{out}$, at trial 20, for the suboptimal network. Alignment between step-2 representation and output weight vector still monotonically encodes rank, but with a flatter profile than in the standard network (see Figure 7).

bioRxiv preprint doi: https://doi.org/10.1101/2023.07.27.550739; this version posted July 28, 2023. The copyright holder for this preprint (which was not certified by peer review) is the author/funder, who has granted bioRxiv a license to display the preprint in perpetuity. It is made available under aCC-BY 4.0 International license.

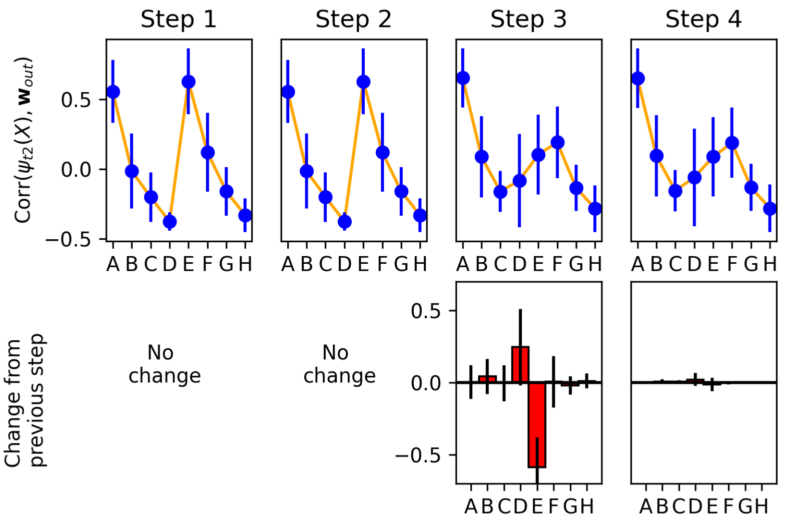

Figure S10: Same as Figure 8, but for the suboptimal network. Representation changes occur at time step 3 (reward delivery) rather than time step 4, and only for the items shown in the current trial.

Figure 7 shows that this alternative solution still maps each single item to a learned representation that encodes its rank, though with a somewhat flatter curve near the middle of the series. As shown in Figure S11, the network still exhibits a symbolic distance effect, confirming that the overall representational scheme is similar. However, performance in list-linking conditions is at or below chance.

What might cause this deficit in list-linking? In Figure S10, we see that representation changes at a given trial occur at reward time only. Importantly, representations are only shifted for the items presented at the current trial. This explains failure of list-linking (which requires transfer of information from currently shown items to other not-shown items).

As mentioned in the main text, almost all meta-trained networks followed one of the two solutions described in the present paper, up to mutually compensating sign changes that leave the fundamental mechanism invariant. The balance of the two solutions may change according to various experimental choices (for example, forcing **A** to contain only positive values increased the proportion of suboptimal solutions). As shown in Figure 3A, the process as described inthe Methods greatly favours the higher-performance cognitive solution over the passive one.

bioRxiv preprint doi: https://doi.org/10.1101/2023.07.27.550739; this version posted July 28, 2023. The copyright holder for this preprint (which was not certified by peer review) is the author/funder, who has granted bioRxiv a license to display the preprint in perpetuity. It is made available under aCC-BY 4.0 International license.

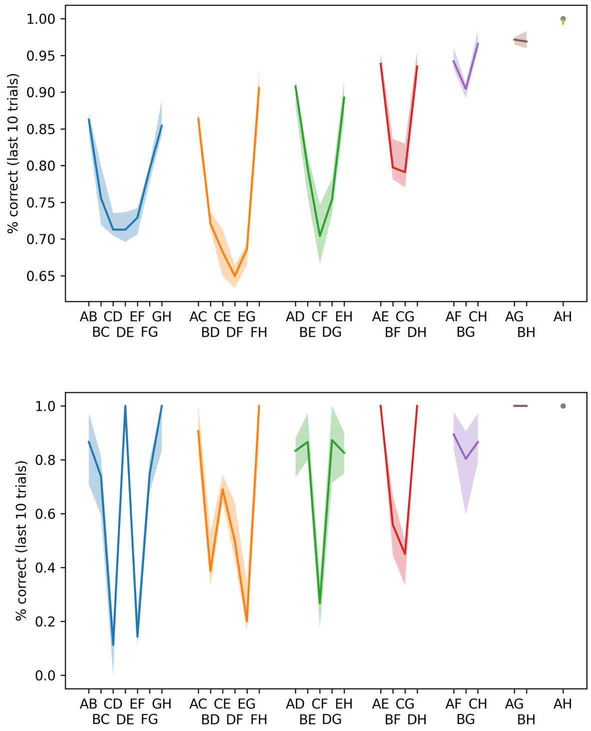

Figure S11: Performance at test time grouped by pairs, in normal conditions(top) and list-linking conditions (bottom). The suboptimal network is still able to produce a symbolic distance effect in normal conditions, but fails the list-linking task. Conventions as in Figure 3 and 4.

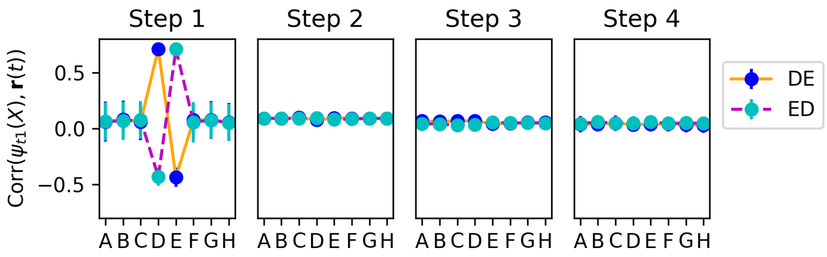

Figure S12: Same as Figure 9, but using ψ_{t1} (the original step-1 feedforward representations of each item) instead of $\tilde{\boldsymbol{\psi}}_{t1}$ (the adapted heterogeneity-aware representations, found by optimization). Again, only runs in which trial 20 showed pair DE or ED are shown. Unadapted, original step-1 representations are strongly present at step 1 (unsurprisingly), but not at any other step.

bioRxiv preprint doi: https://doi.org/10.1101/2023.07.27.550739; this version posted July 28, 2023. The copyright holder for this preprint (which was not certified by peer review) is the author/funder, who has granted bioRxiv a license to display the preprint in perpetuity. It is made available under aCC-BY 4.0 International license.

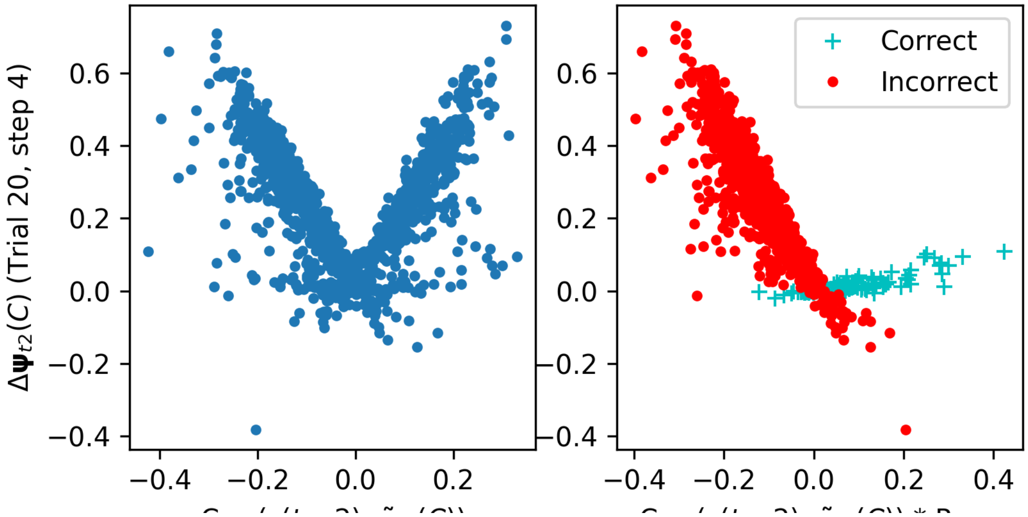

Figure S13: Left: Signed amplitude of reinstated vector $\tilde{\boldsymbol{\psi}}_{t1}(C)$ at time step 2 predicts representation change for C at time step 4, in trial 20 (with pair DE or ED shown as stimulus for this trial). Right: Same data but multiplying x by the sign of the response given for this trial, with separate coloring for correct and incorrect responses. Data contains $\tilde{1}000$ runs with DE not shown before trial 20 (leading to incorrect responses) and $\tilde{1}00$ trials in which it was (leading to mostly correct responses).
